# Does working memory training promote the use of strategies on untrained working memory tasks?

**DOI:** 10.3758/s13421-014-0410-5

**Published:** 2014-04-19

**Authors:** Darren L. Dunning, Joni Holmes

**Affiliations:** 1 Norwich Medical School, University of East Anglia, Norwich Research Park, Norwich, NR4 7TJ UK; 2 MRC Cognition and Brain Sciences Unit, Cambridge, UK

**Keywords:** Working memory, Working memory training, Strategies, Short term memory

## Abstract

Adaptive computerized training has been associated with significant enhancements in untrained working memory tasks, but the nature of the cognitive changes that underpin these improvements are not yet fully understood. Here, we investigate the possibility that training stimulates the use of memory-related strategies. In a randomized controlled trial, participants completed four tests of working memory before receiving adaptive working memory training, nonadaptive working memory training with low memory loads, or no training. Open-ended interviews about strategy use were conducted after the administration of untrained working memory tasks at two time points. Those in the adaptive and nonadaptive groups completed the assessments before (T1) and after (T2) 10 training sessions. The no-training group completed the same set of tasks at T1 and T2, without any training between assessment points. Adaptive training was associated with selective improvements in untrained tests of working memory, accompanied by a significant increase in the use of a grouping strategy for visuospatial short-term memory and verbal working memory tasks. These results indicate that training-related improvements in working memory may be mediated by implicit and spontaneous changes in the use of strategies to subsegment sequences of information into groups for recall when the tasks used at test overlap with those used during training.

## Introduction

Working memory, the cognitive system responsible for the temporary maintenance and processing of information during complex cognitive activities, is important for many everyday functions. including reading comprehension, mental arithmetic, following instructions, and reasoning (Adams & Hitch, 1997; Cain, Oakhill, & Bryant, 2004; Gathercole, Durling, Evans, Jeffcock, & Stone, 2007; Oberauer, Suß, Wilhelm, & Wittmann, 2008). There is accumulating evidence that it can be enhanced by intensive computerized training (see Klingberg, 2010). This is of great potential benefit to individuals with poor working memory skills who struggle to maintain attention and are at risk of educational difficulties (e.g. Gathercole & Alloway, 2008; Kane et al., 2007). Although gains in untrained tests of working memory are commonly reported, the nature of the cognitive changes that underpin these improvements are not yet fully understood. The study reported here investigated the possibility that training encourages the use of strategies that allow individuals to use their existing memory capacities optimally on untrained tests of working memory that share similar task features with the trained tasks.

Working memory training typically involves an individual practicing for multiple sessions on a variety of computer-based working memory tasks that adapt to match current performance. Although there is some debate about the generalizability of training gains (see Gathercole, Dunning, & Holmes, 2012; Melby-Lervåg & Hulme, 2012; Shipstead, Redick, & Engle, 2012), substantial and enduring improvements in untrained working memory tests have been reported in both developmental and adult populations (Chein & Morrison, 2010; Dunning, Holmes, & Gathercole, 2013; Klingberg et al., 2005).

There are two possible processes that might lead to changes on untrained memory tasks. The first is that training increases working memory capacity, enabling participants to store more information. By this account, repeated practice at capacity limits induces long-term plasticity in the brain regions that serve working memory and should, therefore, benefit any activity that calls on the same underlying brain networks (Dahlin, Bäckman, Stigsdotter Neely, & Nyberg, 2009). Both increases and decreases in brain activity have been reported following training (Olesen, Westerberg, & Klingberg, 2004; Westerberg & Klingberg, 2007). Klingberg (2010) states that increases in activity reflect neural plasticity, while decreases, which typically occur more often over shorter training periods (<3 h), reflect strategy development. Despite these changes in neural activity, the benefits of training typically extend only to other working memory tasks that share the same surface features as the trained activities. There is little evidence from methodologically rigorous studies for broader transfer to increases in performance on tasks that load working memory but share little overlap with the structure or materials of the training tasks (e.g., Dunning et al., 2013), which would be expected if training were inducing fundamental changes in brain function.

The second hypothesis is that intensive training encourages more efficient use of existing working memory resources through promoting the development of compensatory strategies to either overcome areas of weakness or capitalize on existing strengths (Holmes, Gathercole, & Dunning, 2009). Strategies are mentally effortful, goal-directed processes that have been shown to enhance working memory performance. One of the most rudimentary of these is rote rehearsal, which involves the repetition of to-be-remembered information (Baddeley, 1999; Rodriguez & Sadoski, 2000; Turley-Ames & Whitfield, 2003). Common strategies include chunking, which is the acquisition of long-term representations of sets of items, and grouping, which describes the volitional subsegmentation of individual memory items into fewer groups (Black & Rollins, 1982; Broadbent, 1975; Cowan, 2001; Lange & Pierce, 1992). Other strategies include visualization (de la Iglasia, Buceta, & Campos, 2005; McNamara & Scott, 2001; Turley-Ames & Whitfield, 2003), the inhibition of irrelevant information (Rosen & Engle, 1998), and the retention of to-be-remembered information by semantic linkage, such as the creation of a story or meaningful links between items (Bower & Clark, 1969; McNamara & Scott, 2001; Turley-Ames & Whitfield, 2003).

Strategy use is associated with efficient working memory function (Baddeley, 2000; Dunlosky & Kane, 2007), and individuals with high memory spans use strategies more than individuals with low spans do (Dunlosky & Kane, 2007; Engle, Cantor, & Carullo, 1992; Friedman & Miyake, 2004; Turley, 1997; Turley-Ames & Whitfield, 2003). According to the strategy mediation hypothesis, variations in strategy use also predict individual differences in span performance. Individuals who use effective strategies, such as grouping and other effortful and demanding algorithms such as chunking and chaining (Dunlosky & Kane, 2007), perform better on span tasks than those who use less effective strategies such as rote rehearsal (McNamara & Scott, 2001). The use of effective strategies also mediates the relationship between working memory span and higher-order cognitive activities such as reading comprehension (Baddeley, 2000; McNamara & Scott, 2001; Salthouse, 1996), but only when identical strategies are afforded by both the memory and cognitive ability tasks (Bailey, Dunlosky, & Kane, 2008; note that other factors, such as processing speed, influence this relationship too).

Training participants explicitly to use strategies facilitates increases in short-term and working memory performance. Improvements in short-term memory (STM) tasks have been reported when adults engage in rehearsal (e.g., Broadley, MacDonald, & Buckley, 1994; Rodriguez & Sadoski, 2000), visualisation (e.g., de la Iglesia et al., 2005), chunking (e.g., Carr & Schneider, 1991; Lange & Pierce, 1992), and chaining (e.g., McNamara & Scott, 2001). Rehearsal, chaining, and visualisation strategies also improve adults’ performance on working memory span tasks (McNamara & Scott, 2001; Turley-Ames & Whitfield, 2003). Similar benefits are seen in children, with enhancement of verbal STM and verbal working memory after computerized training that teaches and encourages the use of rehearsal, visual imagery, and story generation (St. Clair-Thompson, Stevens, Hunt, & Bolder, 2010).

Adaptive working memory training programs do not explicitly teach meta-cognitive techniques, but they may promote the development or enhancement of strategies spontaneously employed to complete working memory tasks. Introspective reports from children in our own training studies support the notion that, even in the absence of direct strategy instruction, repeated practice on working memory tasks promotes the development of idiosyncratic strategies. When asked what they thought had helped them to improve, 37 % of children with low working memory and 67 % of children with ADHD reported using strategies that included rehearsal and visualization after training (Holmes et al., 2009; Holmes et al., 2010). The restricted transfer of training gains to tasks that share the same structures and materials as the training tasks (see Shipstead et al., 2012, for a review) provides further evidence that training may promote the development of strategies for specific task paradigms, rather than inducing changes in the underlying working memory substrate (see von Bastian & Oberauer, 2013).

The aim of the present study was to investigate whether adaptive training encouraged participants to use strategies on untrained working memory tasks. To assess the impact of training, participants were assigned to an adaptive training group, an active control group who completed nonadaptive training, or a no-intervention group who did not undergo training. To investigate whether improvements on these untrained tasks were accompanied by changes in strategy use that were specific to adaptive training, verbal reports of strategy use were compared across the three groups at two time points (before and after training for the adaptive and nonadaptive groups, and at the same two time points for participants in the no-intervention group). Assessments of strategy use were not obtained during training to avoid influencing participants’ approach to training. It was predicted that adaptive training would be associated with significant increases in performance on untrained memory tasks and that these changes would be accompanied by changes in the strategies participants reported using to complete the tasks posttraining.

## Method

### Participants and procedure

A total of 45 undergraduate students 18–21 years of age with fluent spoken and written English skills were recruited. Participants were randomly assigned to one of three conditions: an adaptive training group (*n* = 15; 14 females; mean age = 20 years 3 months, *SD* = 17.92 months), a nonadaptive training group (*n* = 15; 12 females; mean age = 20 years 1 month, *SD* = 14.32 months), or a no-training group (*n* = 15; 10 females; mean age = 20 years 3 months, *SD* = 18.73 months). Participants were paid an equal amount for participation, irrespective of group assignment.

All participants completed a set of pretraining assessments (T1). Those in the adaptive and nonadaptive groups then trained for 10 sessions before completing a set of posttraining assessments (T2). The no-training group completed the same set of tasks at T2, without any training between assessment points. All assessments were conducted by a research assistant blind to group status. Two participants, one in each of the adaptive and nonadaptive training groups, failed to complete training. Therefore, the final numbers of participants included in the analyses were adaptive training group, *n* = 14; nonadaptive training group, *n* = 14; and no-intervention group, *n* = 15. Post hoc statistical power analysis for the group × time interactions was .83 for a medium effect size, with a *p* level of .05 (Erdfelder, Faul, & Buchner, 1996).

The study was approved by the University Ethics Committee, and written consent for participation was obtained from all participants prior to study commencement.

### Materials

#### Working memory assessments

Participants completed four subtests of the Automated Working Memory Assessment (AWMA; Alloway, 2007) at T1. The same four subtests of were readministered at T2. Digit recall, which required the immediate serial recall of a list of verbally presented digits, was used to assess verbal STM. Dot matrix was used to assess visuospatial STM. This required participants to recall a series of visually presented dots on a 4 × 4 grid in the correct order. Verbal working memory was measured using the backward digit recall subtest, in which participants were required to recall a series of verbally presented digits in reverse serial order. Mr X was used to index visuospatial working memory performance. For this subtest, participants were asked to decide whether two figures presented on screen were holding a ball in the same hand as one another. The ball held by the figure on the right could appear at one of one of six possible compass points. Having decided whether the two figures were holding the ball in the same or a different hand, participants were then asked to recall the location of the ball held by the figure on the right. Task difficulty was adjusted by increasing the number of pairs of figures presented, hence increasing the number of locations to be stored. Retest reliability for each of the measures is as follows: digit span, .89; dot matrix, .85; Mr X, .84; backward digit recall, .86 (Alloway, 2007). Standard scores were produced for each test.

#### Working memory training

Participants in the adaptive condition completed 10 sessions of the RM version of Cogmed Working Memory Training (CWMT; Cogmed, 2005). This involved training on a variety of working memory tasks in a computerized game environment for approximately 35 min a day (120 trials) on their home computer over a period of between 2–4 weeks. The trials were equally divided across eight different training tasks, selected from a bank of nine tasks. Participants trained on the same eight tasks for the first 5 days of the training period. On the 6th day, one of the tasks was replaced by the remaining task from the bank. CWMT is a commercially available product, meaning the exercises were delivered in a preset manner each session. Task difficulty adapted to the participant’s current memory span on a trial-by-trial basis. A description of each of the training tasks follows: *visual data link*, where participants were required to recall a series of locations presented in a 4 × 4 grid in serial order; *rotating data link*, which was identical to visual data link, except that the grid rotated clockwise 90^o^ prior to recall; *data room*, where a series of locations were highlighted on the walls, ceiling, and floor of a 3-D room, which participants were asked to recall in serial order; *input module*, where participants were required to recall a series of verbally presented digits in reverse serial order by inputting the numbers into a keypad on screen that remained visible throughout presentation; *input module with lid*, which was identical to input module, except that the keypad was hidden when the digits were spoken aloud and were displayed only for recall; *decoder*, where participants heard a series of letters, which they had to recall in correct serial order by selecting the correct letter for each position in the sequence from a choice of three; *rotating dots*, where a sequence of lights lit up one at a time on a dial that was rotating clockwise and participants were required to recall the serial order of the lights, while the dial continued to rotate; *numbered grid*, where numbers appeared in a random order in different locations on a grid and participants were required to recall both the sequence of digits in ascending order and the correct location of the numbers; *random letters*, where a series of letters were presented verbally and, as each letter was presented, a random location lit up on a dial; a target letter then appeared in the middle of the dial that matched one previously presented, and participants were required to recall the location that lit up when the target letter was heard.

Participants in the nonadaptive group trained on a placebo version of the program that was identical to CWMT, except that the tasks were set at a low span level of two throughout the training period with no increase in difficulty.

Remote supervision of training was achieved using the Cogmed Training Web. This is an online resource that allowed the researchers to review and monitor the results of training and to ensure that all training sessions were completed.

#### Strategy use interviews

Retrospective global reports of strategy use were obtained from participants immediately after each of the four working memory subtests at both T1 and T2. Participants were first asked whether they had used a strategy to complete the memory task. If they answered in the affirmative, they were asked to elaborate, in as much detail as possible, how they had tried to remember the information and to think about what strategies they had used. Participants were given as much time as they needed to provide their responses, which were recorded and transcribed after testing. The written reports were independently categorized into strategy types by two raters who were briefed about strategies beforehand. These category types were taken from the research literature on strategy use and included rehearsal, grouping, visualisation, imagery, inhibiting irrelevant information, chunking, and chaining (e.g., Dunlosky & Kane, 2007; Turley-Ames & Whitfield, 2003). If a strategy reported by a participant was not covered by an existing strategy type, a new type was created. Agreement between the raters was >90 %, and In the event of discordant experimenter coding, participant responses were reexamined by a third rater to reach a consensus.

## Results

### Working memory

**Fig. 1:**
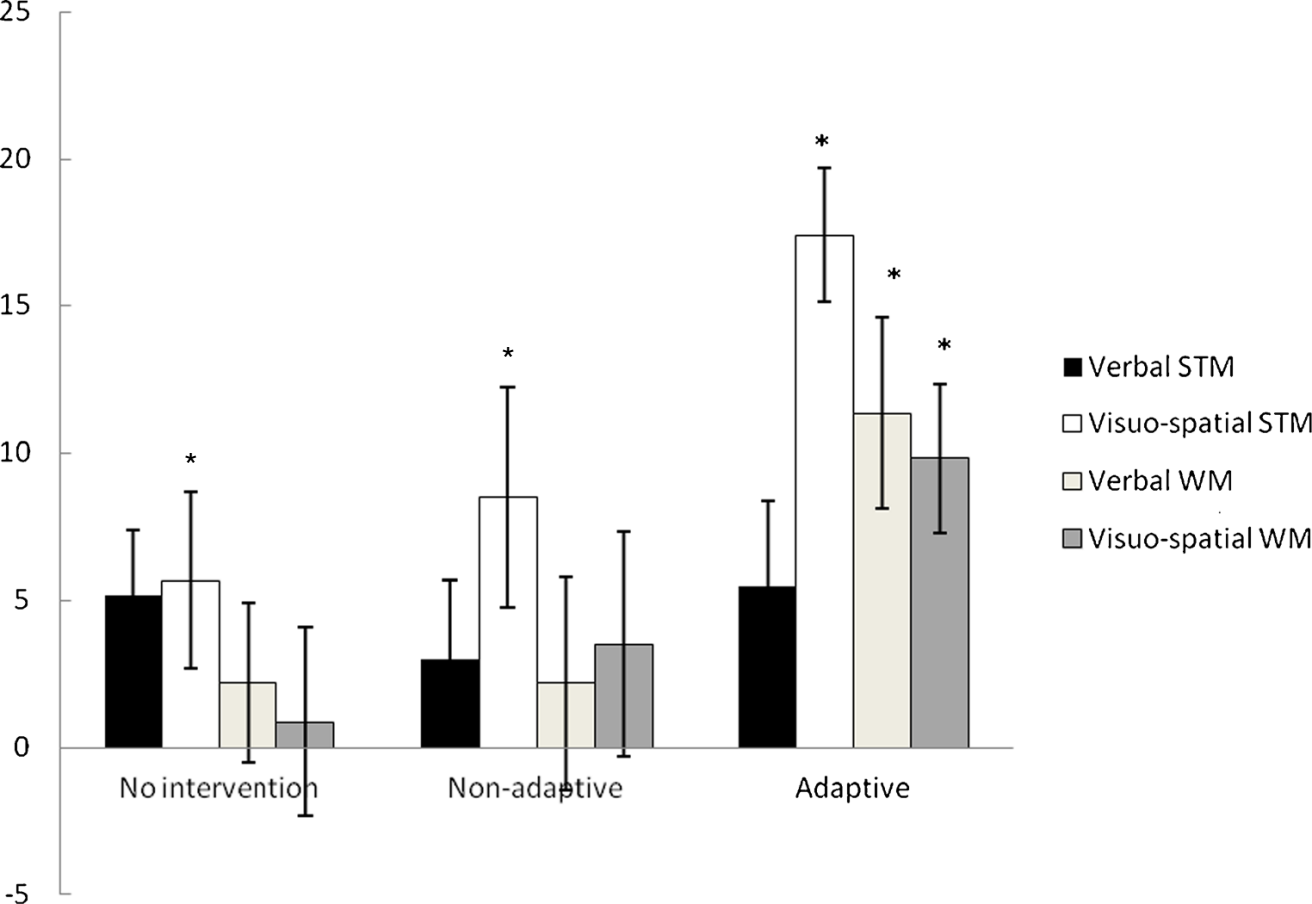
Changes in standard scores on the Automated Working Memory Assessment pre- to posttest, by group. STM, short-term memory; WM, working memory. *Significant change pre- to posttest

Figure 1 displays changes in standard scores before and after training for all four aspects of working memory, for each of the three groups. There were no significant differences between the groups at baseline on any of the measures (all *p*s > .05). Paired-sample *t*-tests established significant gains from T1 to T2 in visuospatial STM and verbal and visuospatial WM for the adaptive training group (Cohen *d*s = 1.47, 0.87, and 0.73, respectively). There was also a significant increase in visuospatial STM for the no-intervention and nonadaptive groups (Cohen’s *d*s = 0.51 and 0.74, respectively).

Group (adaptive, nonadpative, no intervention) × time (T1, T2) ANOVAs established significant interactions for visuospatial STM, *F*(1, 39) = 4.47, *p* =.018, *η* = .186, verbal working memory, *F*(1, 39) = 3.49, *p* = .04, *η* = .152, and visuospatial working memory, *F*(1, 39) = 3.12,*p* = .05, *η* = .138. Gains were significantly greater for the adaptive group than for both control groups for visuospatial STM and working memory (*p*s < .05). The group × time interaction was not significant for verbal STM, *F*(1, 39) = 0.242, *p* = .786, *η* = .012.

### Strategy use

The frequency with which each type of strategy was used before and after training was analyzed by group in a series of chi-square tests. Percentages of participants using different strategy types pre- and posttraining for the verbal and visuospatial STM and working memory tasks are shown in Table 1.

**Table 1:** Number of participants (%) using different strategy types to complete memory tasks pre- and posttraining

		Nonadaptive						Adaptive					No Intervention			
		Pre	Post	*χ*2	*p*	*V*	Pre	Post	*χ*2	*p*	*V*	Pre	Post	*χ*2	*p*	*V*
Verbal short-term memory	Rehearsal	14.29	14.29	0.00	1.00	.00	21.43	21.43	0.00	1.00	.00	13.33	20.00	1.78	.25	.09
	Semantic	7.14	0.00	7.25	.01	.19	14.29	0.00	15.54	.00	.27	6.67	6.67	0.00	1.00	.00
	Visualization	14.29	28.57	6.66	.15	.18	14.29	0.00	15.54	.00	.27	13.33	20.00	1.78	.25	.09
	Grouping	21.43	28.57	1.71	.25	.09	28.57	71.43	35.28	.00	.42	26.67	26.67	0.00	1.00	.00
	Rhythm		7.14	7.14	0.00	1.00	.00	7.14	7.14	0.00	1.00	0.00	0.00	0.00		
	Phonetically	7.14	7.14	0.00	1.00	.00	0.00	0.00	0.00	1.00	.00	6.67	0.00	7.25	.01	.19
	Imagery	0.00	0.00				0.00	0.00				6.67	0.00	7.25	.01	.19
Visuospatial short-term memory	Rehearsal	7.14	21.43	8.14	.01	.20	7.14	14.29	2.07	.17	.11	13.33	6.67	2.00	.24	−.10
	Semantic	7.14	0.00	7.25	.01	.19	7.14	7.14	0.00	1.00	.00	6.67	6.67	0.00	1.00	.00
	Visualization	57.14	35.71	8.86	.00	.21	64.29	28.57	24.62	.00	.35	60.00	53.33	1.00	.39	.07
	Grouping	0.00	0.00				0.00	50.00	66.67	.00	.58	6.67	13.33	2.00	.24	.10
	Imagery	0.00	7.14	7.25	.01	.19	0.00	0.00				0.00	0.00			
	Concentrate	0.00	7.14	7.25	.01	.19	0.00	0.00				0.00	0.00			
	Verbal recoding	0.00	7.14	7.25	.01	.19	14.29	21.43	1.70	.26	.09	0.00	6.67	7.25	.01	.19
Verbal working memory	Rehearsal	14.29	14.29	0.00	1.00	.00	35.71	42.86	1.03	.39	.07	20.00	26.67	1.36	.32	.05
	Semantic	7.14	0.00	7.25	.01	.19	7.14	0.00	7.25	.01	.19	0.00	0.00	0.00	1.00	.00
	Visualization	28.57	35.71	1.12	.37	.08	21.43	14.29	1.70	.26	.09	26.67	46.67	8.85	.01	.21
	Grouping	7.14	14.29	2.61	.17	.11	7.14	78.57	105.75	.00	.73	13.33	13.33	0.00	1.00	.00
	Rhythm	7.14	0.00	7.25	.01	.19	0.00	7.14	7.25	.01	.19	6.67	0.00	7.25	.01	.19
	Phonetically	7.14	0.00	7.25	.01	.19	0.00	0.00				13.33	0.00	13.90	.00	.26
	Imagery	0.00	0.00				0.00	0.00				6.67	0.00	7.25	.01	.19
	Concentrate	0.00	7.14	7.25	.01	.19	7.14	0.00	7.25	.01	.19	0.00	0.00			
	Speed	0.00	0.00				0.00	7.14	7.25	.01	.19	0.00	0.00			
Visuospatial working memory	Rehearsal	0.00	7.14	7.25	.01	.19	0.00	0.00				6.67	6.67	0.00	1.00	.00
	Semantic	14.29	7.14	2.61	.17	.11	21.43	14.29	1.70	.26	.09	20.00	13.33	1.78	.25	.09
	Visualization	28.57	21.43	1.71	.25	.09	14.29	14.29	0.00	1.00	.00	26.67	20.00	1.36	.32	.08
	Chunking	0.00	0.00				0.00	0.00				0.00	13.33	13.90	.00	.26
	Imagery	7.14	7.14	0.00	1.00	.00	0.00	0.00				0.00	0.00			
	Concentrate	0.00	0.00				7.14	0.00	7.25	.01	.19	6.67	0.00	7.25	.01	.19
	Verbal recoding	0.00	0.00				7.14	0.00	7.25	.01	.19	0.00	0.00			
	Inhibiting	0.00	0.00				0.00	0.00				6.67	0.00	7.25	.01	.19

*Note. V* = Cramer’s *V* effect sizes: .1 = small, .3 = medium, .5 = large. Statistics not performed when strategy not reported at pre- or posttest

There was a significant increase in the number of participants in the adaptive group who reported using grouping as a strategy to complete the verbal STM, visuospatial STM, and verbal working memory tasks. These effects were large (Cramer’s *V* ranging .42 to.73), with at least 50 % more participants reporting use of this particular strategy posttraining. Significant group × time interactions for verbal STM, *F*(2, 39) = 3.13, *p* = .05, *η* = .138, visuospatial STM, *F*(2, 39) = 7.561, *p* = .002, *η* = .279, and verbal working memory, *F*(2, 39) = 9.145, *p* = .001, *η* = .3.19, established that a significantly greater number of participants in the adaptive group used grouping at T2 than in both control groups.

To explore further whether changes in the use of grouping were related to gains in working memory, participants in the adaptive group were split into two groups: (1) those who did not use grouping at T1 but reported using it at T2 and (2) those who did not report a change in the use of grouping T1 to T2. Although there were no significant differences in gains between those who reported a change in the use of grouping and those who did not (all *p*s > .05), the Cohen’s *d* effect sizes for the visuospatial STM and verbal working memory tasks were .39 and .59, respectively. Those who used grouping for these tasks posttraining made bigger gains on the tasks: visuospatial STM, *M* = 19.28 (*SD* = 9.48), v *M* = 15.75 (*SD* = 8.53), and verbal working memory, *M* = 13.27 (*SD* = 12.83), v *M* = 6.00 (*SD* = 12.02). The absence of significant effects is likely due to low power, which resulted from dividing the group of 14 participants in the adaptive group into two smaller groups.

There were smaller increases in the percentage of participants in the adaptive group who used rhythm or speed to complete the verbal working memory task at T2 (Cramer’s *V* = .19 in both cases). There were also a number of small yet significant increases in strategy use at T2 in the nonadaptive and no-intervention groups. A greater number of participants in the nonadaptive group reported using rehearsal, imagery, concentration, or verbal recoding for the visuospatial STM task, concentration for the verbal working memory task, and rehearsal for the visuo-spatial working memory task at T2. In all cases, the effect sizes were small, with a maximum increase of 2 participants reporting using the strategy at T2 (all Cramer’s *V*s < .2).

The number of participants in the no-intervention group reporting use of a verbal recoding for the visuospatial STM task, visualization for the verbal working memory task and grouping for the visuospatial working memory task increased at T2. Although significant, these effects were small, with 2 or 3 participants using the strategy following training (all Cramer’s *V*s < .26).

## Discussion

These findings established that adaptive working memory training was associated with significant improvements in untrained tests of working memory in young adults. These improvements were accompanied by changes in the strategies used to complete the tasks.

Observing improved working memory performance in an adult group demonstrates that cognitive training improves performance on unpracticed memory tasks for individuals whose baseline performance is in the age-appropriate range. Research studies of working memory training typically use a 20-session protocol (e.g., Dunning et al., 2013; Klingberg et al., 2005). Here, we have shown for the first time, using a double-blind RCT, that just 10 training sessions are sufficient to boost performance. The sustainability and generalizability of these gains is yet to be seen, but these results provide preliminary evidence that shorter training regimes may be effective. This is particularly useful for future experimental manipulations of training effects where it is not always feasible to administer 15 + h of intervention.

Adaptive training was associated with selective gains in tests of visuospatial STM and verbal and visuospatial working memory. These results reinforce the outcomes of a recent RCT with children with low working memory in which training also failed to boost performance on a verbal STM task (Dunning et al., 2013). Verbal storage aspects of working memory place minimal demands on executive attention (e.g., Alloway, Gathercole, & Pickering, 2006; Engle, Kane, & Tuholski, 1999). It is therefore possible that training enhances only those aspects of working memory that are executively demanding.

The present study offers important insights into the cognitive changes that might mediate improvements in working memory function following training. A significantly greater number of participants who completed adaptive training reported using a grouping strategy at T2, as compared with those receiving nonadaptive or no training. These data suggest that training gains likely reflect more than just a modification to basic working memory capacities via neural plasticity (e.g., Klingberg, 2010) and that training might stimulate spontaneous strategy use in the absence of direct strategy instruction (Miller, 1990, 1994). It is possible that repeated exposure to memory tasks at the boundaries of capacity limits forces participants to actively reflect on their approach to the activities and, consequently, engage in strategic behavior to improve performance.

According to the strategy mediation hypothesis, in which the use of effective strategies is associated with better performance on working memory span tasks (McNamara & Scott, 2001), training-related increases in memory performance might be mediated by more efficient use of the working memory capacity available. Significant increases in the use of grouping strategies were observed for untrained tests of verbal STM, visuospatial STM, and verbal working memory tasks following training. The changes in strategy use for visuospatial STM and verbal working memory were accompanied by significant increases in performance. Importantly, these two tasks overlap directly with the training activities in terms of both task structure and material. The visuospatial STM test required the serial recall of spatial locations in forward order, and the verbal working memory test the serial recall of digits in reverse order. Likewise, multiple training activities required the immediate recall of spatial information in the order in which it was presented or the backward recall of digit strings. Despite reported increases in the use of grouping for the verbal STM task, there were no selective improvements in performance on this task for the adaptive group. This could reflect participants’ enhanced awareness of the use of grouping strategies for serial recall tasks during training, which they erroneously ascribed to the verbal STM task at test. Although the verbal STM test (digit recall) overlaps with the trained activities in terms of the requirement for serial recall, it differs in terms of the combination of materials and task demands: There was no training task that explicitly required the immediate serial recall of digit lists. Thus, here the combination of both increases in performance and changes in the use of grouping is specific to untrained tests that directly overlap with the trained tasks. This specific transfer of strategy knowledge from the trained tasks to new versions of the same paradigm suggests that training may be promoting the development of highly task-specific strategies that enhance the use of existing working memory resources (e.g., von Bastian & Oberauer, 2013).

An alternative possibility is that increases in capacity that arise from training afford the use of effective strategies. The strategy-as-effect hypothesis supposes that all individuals are equally strategic across unchallenging tasks but that, for demanding tasks, having a higher working memory capacity affords the cognitive capacity to produce and implement effortful strategies while concurrently performing the task (Dunlosky & Kane, 2007; Dunlosky & Thiede, 2004). In training studies, low-span individuals are able to benefit only from instruction for less effective and low effort strategies such as rehearsal. Even with explicit instruction, they are unable to use demanding strategies such as grouping and chaining successfully, presumably because their capacity is already taxed to its limit (Turley-Ames & Whitfield, 2003). An increase in the use of grouping for both verbal and visuospatial tasks in the present study suggests that the utilization of domain-general effective strategies is enhanced through training, which might reflect an increase in the availability of domain-general executive resources for strategic deployment that occurs as memory capacity increases.

There is currently limited evidence (e.g., Salminen, Strobach, & Schubert, 2012) from methodologically rigorous studies that training-induced changes in working memory extend beyond enhanced performance on other working memory tasks (Shipstead et al., 2012). In an RCT with children, we found that substantial gains in working memory did not translate into improvements in either analogues of working-memory-demanding classroom activities or measures of academic ability (Dunning et al., 2013). RCTs with adults have demonstrated similar effects (Chein & Morrison, 2010; Dahlin et al., 2009). The strategy affordance hypothesis posits that the relationship between memory span and other cognitive tasks is mediated by strategy use *only* when both tasks require use of the same strategies (e.g., Bailey et al., 2008). We suspect that the absence of transfer to other tasks and situations that load working memory arises because training promotes the development of highly task-specific strategies that do not overlap with those used outside structured working memory span tasks. By this account, the consistent and substantial gains observed on a variety of untrained working memory tasks (e.g., Dunning et al., 2013; Holmes & Gathercole, 2013; Holmes et al., 2009; Holmes et al., 2010) might arise because they afford the same strategies as the training tasks.

If existing interventions, which train performance on rarefied laboratory-style working memory tasks, are training highly task-specific strategies, they are unlikely to remediate the everyday consequences of poor working memory. To bridge the gap between the specific cognitive gains induced by training and their flexible application to other working-memory-demanding situations, existing programs may need to be modified to provide adaptive training that encourages the recruitment of strategies across a variety of tasks that map more directly onto the challenging cognitive situations in which working memory is used in everyday life. Alternatively, posttraining practice in applying newly developed strategies to complex cognitive situations might prove fruitful. Training might also produce broader generalization when it effectively *discourages* the use of task-specific strategies—for example, by placing heavier demands on domain-general processes that might incite broader transfer (e.g., attention control, updating, and interference resolution). Indeed, there is already some promising evidence that training the ability to switch mental sets results in broad enhancements of executive attention, which arise as a consequence of the sustained demands of maintaining fast-changing goals and continuously suppressing recently relevant cues as response sets change (Karbach & Kray, 2009).

In summary, this establishes that adaptive working memory training can boost performance on untrained working memory tests in typically developing healthy young adults and that changes in performance following training may be mediated, at least in part, by increases in the use of grouping. Of course, there were gains for some participants without reported changes in strategy use. This likely reflects that there is more than one route to improved performance or that training induces subtle changes in other idiosyncratic strategies that are less easy to articulate. The retrospective report method used in this study provided rich, detailed descriptions of the types of strategies participants used. However, the general nature of these reports may have allowed for forgetting to occur, for individuals to draw upon how they believed they should have completed the task rather than how they actually did, or for participants to draw on the examples of strategies they used for only a few trials or in training (Dunlosky & Kane, 2007). As such, the reports gathered here do not provide definitive evidence that strategy use accounts for variation in training-related improvements in working memory performance, but they do provide an interesting perspective to stimulate further work. Experimental studies that control the strategies participants are able to employ—for example, by manipulating the presentation rate or semantic links between the stimuli presented during training—will provide exciting future investigations in this direction.
